# Renal tuberculosis and iliopsoas abscess: Two case reports

**DOI:** 10.3892/etm.2014.1623

**Published:** 2014-03-14

**Authors:** HONG-LAN WEI, LEI WANG, XING-GUO DU, YANG WU, HUA LI, YUAN CAI, XIAO-HONG SONG, CHENG-XU LI, LI-PING DONG, ZHI-FEN LIU, XIA ZHAO, JUN-WU DONG

**Affiliations:** Department of Nephrology, Puai Hospital, Tongji Medical College, Huazhong University of Science and Technology, Wuhan, Hubei 430033, P.R. China

**Keywords:** renal tuberculosis, iliopsoas abscess

## Abstract

The urinary system is the second most commonly affected site of extrapulmonary tuberculosis (TB). Due to the diverse and atypical clinical manifestations of urinary TB, the disease is easy to misdiagnose. In the present study, two cases of renal TB are reported, which had completely different clinical manifestations. The first case is a female who presented with loin pain and fever. Purified protein derivative (PPD) and TB antibody tests were negative and computed tomography (CT) scans showed a low density focus in the right kidney with an iliopsoas abscess. The typical CT findings indicated renal tuberculosis. Anti-TB drugs were effective proved the diagnosis. The second case is a male who presented with intermittent gross hematuria. Acid-fast bacilli in urine and TB antibody tests were positive. CT scans revealed a low density focus in the unilateral kidney with a slight expansion of the pelvis, calices and ureter. The patients were treated with the anti-TB drugs and the clinical manifestations disappeared. The diagnosis of urinary TB is challenging in certain cases; when there is no response to the usual antibiotics in patients with fever or gross hematuria, TB should be suspected. CT is the mainstay for investigating possible urinary TB.

## Introduction

In developing countries, tuberculosis (TB) is common and may involve any system, including the respiratory, gastrointestinal, cardiac, central nervous, musculoskeletal and genitourinary systems. The data from World Health Organization shows that in 2010, there were between 8.5 and 9.2 million cases of TB globally, equivalent to 128 cases per 100,000 of the population (1). The greatest amount of the estimated number of cases occurred in Asia (59%) and Africa (26%) (1). Pulmonary TB is the most common. The urinary system has been reported to be the second most commonly affected site of extrapulmonary TB (2). Due to diverse and atypical clinical manifestations, urinary TB is easy to misdiagnosis. The positive diagnostic rates of acid-fast bacilli in urine, intravenous excretory urograms (IVUs) and B ultrasounds are limited. Computed tomography (CT) scans are feasible and the mainstay for investigating possible urinary TB. In the present study, two cases of renal TB with markedly different clinical manifestations and CT features are reported. Informed consent was obtained from the patients. THe study was approved by the Biological and Medical Ethics Committee of Puai Hospital, Tongji Medical College, Huazhong University of Science and Technology (Wuhan, China).

## Case reports

### Case 1

**Figure 1:**
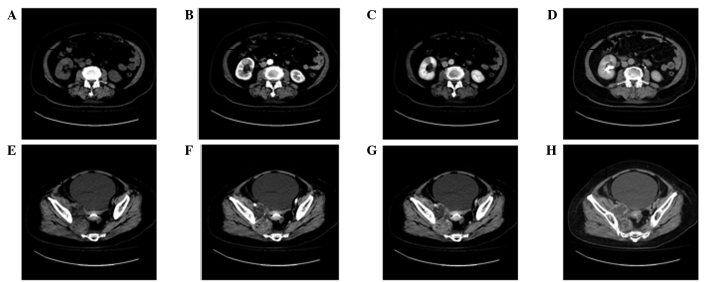
Case 1. Contrast-enhanced CT scans show a low density focus (1.9×2.1 cm) in the right kidney and an iliopsoas abscess. (A and E) Plain scan; (B and F) arterial phase; (C and G) venous phase; and (D and H) delayed phase. CT, computed tomography.

**Figure 2:**
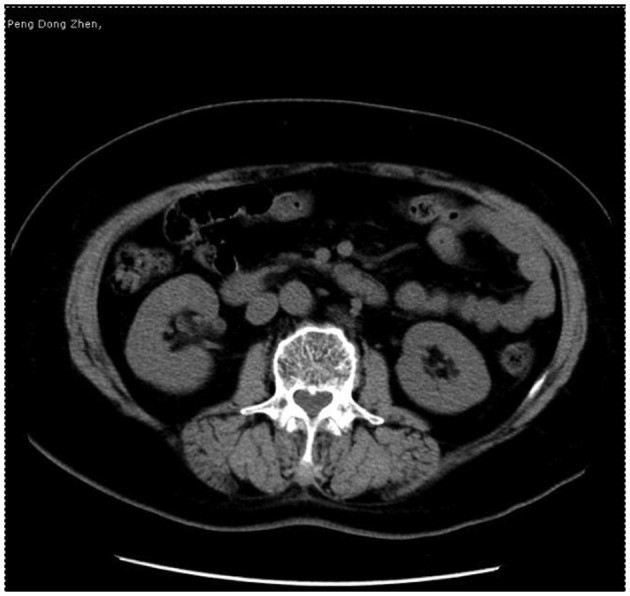
Case 1. CT plain scan shows the disappearance of the low density focus and the iliopsoas abscess. CT, computed tomography.

A 63-year-old female was admitted to the Department of Nephrology at the Puai Hosipital (Wuhan, China) with symptoms of pain in the right loin for two months and fever for one day. The patient had not experienced coughing, hemoptysis, weight loss, night sweats or TB contact, but did have a history of hypertension and diabetes mellitus. There was no past or family history of TB. The patient had a temperature of 39.0°C, a blood pressure of 186/84 mmHg and tenderness of the right loin. Laboratory results showed a white blood cell (WBC) count of 20.13×10^9^/l (84.5% neutrophils and 9.9% lymphocytes) and hemoglobin and platelet levels were normal. Urine tested positive for WBCs, and no urinary protein or microscopic hematuria was detected. Liver and renal functions were normal. Purified protein derivative (PPD) and TB antibody tests were negative. *Staphylococcus aureus* growth was observed in blood culture and *Enterococcus faecalis* growth was observed in uric culture. Other serological tests for antinuclear antibodies, rheumatoid factor and HIV were negative. Chest X-ray and abdominal ultrasound observations were normal. Empiric antibiotic therapy of intravenous linezolid, norvancomycin and imipenem was administered but was not successful. Symptoms of pain in the right loin and fever remained. An abdominal CT scan was then performed, which identified a low density focus (1.9×2.1 cm) in the lower pole of the right kidney and an iliopsoas abscess (Fig. 1). On the basis of clinical and laboratory observations, renal TB and iliopsoas abscess were suspected. The patient was treated with the anti-TB agents isoniazid (Xinyi, Shanghai, China), rifampicin (Yanan, Shanghai, China) and ethambutol (Hongqi, Shenyang, China). One week later, the body temperature had decreased to normal and the pain had alleviated. After two months, repeated abdominal CT scans were performed and the low density focus and iliopsoas abscess had disappeared (Fig. 2).

### Case 2

**Figure 3:**
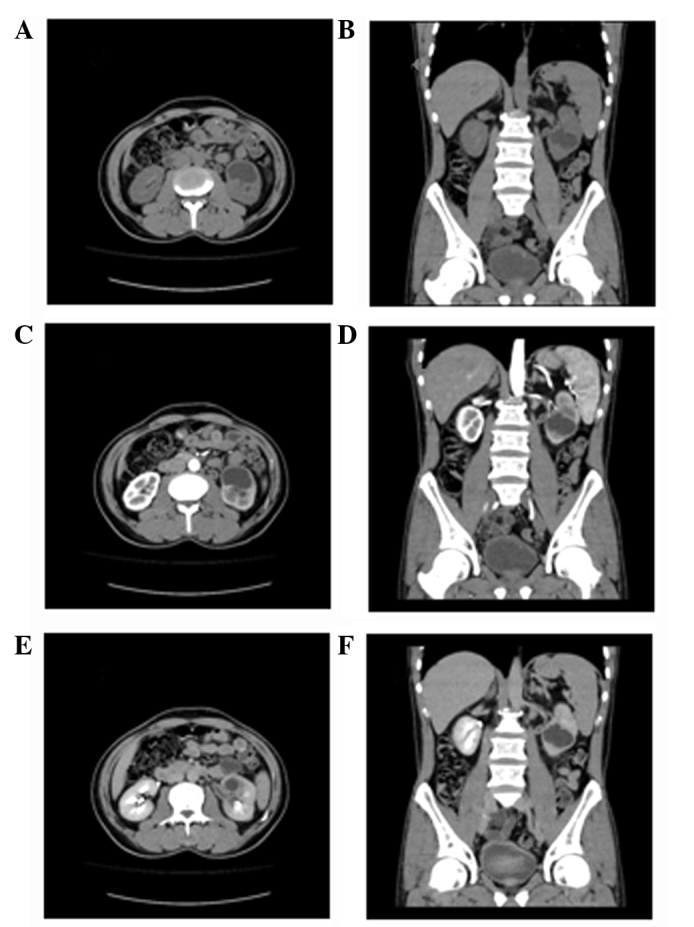
Case 2. CT scans show a low density focus (3.7×3.3cm) in the left kidney with a slight expansion of the pelvis, calices and ureter. (A and B) Plain scan; (C and D) arterial phase; and (E and F) delayed phase. CT, computed tomography.

A 53-year-old male presented with intermittent gross hematuria for three months and left loin pain for two months. A presumptive diagnosis of kidney calculi was made. The patient was treated in a local hospital with antibiotics, which were ineffective. The patient was admitted to the Department of Nephrology at the Puai Hospital (Wuhan, China). The patient had a history of diabetes mellitus, but no past or family history of TB. Left renal area percussion pain was noted during physical examination. Clinical tests had the following results: WBC total count, 6.5×10^9^/l (70.3% neutrophils); hemoglobin, 117 g/l; serum urea, 10.16 mmol/l; serum creatinine, 120.1 μmol/l; serum uric acid, 400.2 μmol/l; serum calcium, 1.93 mmol/l; serum phosphorus, 0.88 mmol/l; serum carbon dioxide, 20.9 mmol/l; and erythrocyte sedimentation rate (ESR), 36 mm/h. Urinary WBC, urine protein and microscopic hematuria tests were positive. TB antibody [16 kDa, lipoarabinomannan (LAM) and 38 kDa] tests were positive and acid-fast bacilli were detected in the urine. CT scans revealed a low density focus (3.7×3.3 cm) in the left kidney with a slight expansion of the pelvis, calices and ureter (Fig. 3). Urinary TB was suspected and the patient was treated with anti-TB drugs for six months. Following the treatment, the gross hematuria disappeared and the loin pain was alleviated.

## Discussion

The common manifestations of TB are fever, weight loss and night sweats. However, in urinary TB these are unusual. The clinical manifestations of urinary TB are nonspecific, including back, flank and suprapubic pain, hematuria, increased urinary frequency and nocturia, which may also indicate a conventional bacterial urinary tract infection (3). In a study of 31 subjects with genitourinary TB in Nigeria, 51.6% had fever, 22.6% had dysuria and others had back, loin or abdominal pain/tenderness (4). TB should be suspected particularly with sterile pyuria or when there is no response to the usual antibiotics (3). In the first case in the present study, the patient had a fever. In addition, uric and blood cultures were positive, but the bacterium differed. It was presumed that one or both were contamination.

Based on the presence or absence of underlying disease, iliopsoas abscesses may be classified into primary (30% cases) or secondary (70% cases) (5). The most common origin of primary iliopsoas abscesses is *Staphylococcus aureus* (88%). Other organisms, including *Streptococci* (5%) and *Escherichia coli* (3%) may also be involved (6). A primary iliopsoas abscess is caused by the hematogenous or lymphatic spread of bacteria, while a secondary iliopsoas abscess is likely to occur from the direct spread of infection from an adjacent structure (7,8). All the major abdominal and pelvic structures are in close contact with the iliopsoas muscle; therefore, any infection in these structures is able to spread to the iliopsoas muscle. Gastrointestinal diseases, genitourinary problems, femoral vessel catheterization, vertebral osteomyelitis and endocarditis may all lead to secondary ilioposoas abscesses (5). In a series of 124 patients with secondary iloposoas abscesses, the most common origin was skeletal infection (50.5%), followed by alimentary tract (24.8%) and renal infections (17.5%) (8). In the first case of the present study, an ilioposoas abscess was identified. As there was a low density focus in the right kidney, it was suspected that the ilioposoas abscess was secondary.

The clinical manifestations of renal TB are nonspecific and biopsy of the kidney or abscess is invasive. In one study, the positive diagnostic rate of acid-fast bacilli in urine sedimentation was 42.7% and the positive diagnostic rates of IVU and B ultrasound were 69.1 and 28.3%, respectively (9). CT is the mainstay for investigating possible urinary TB and has demonstrated a positive diagnostic rate of 84.3% (9). Features of renal TB in CT are multiple and complex. In the early stages of the disease, CT plain scans manifest as a single low density focus with edge blur, which has a significantly lower density than renal tissue in enhanced scans and may mimic malignancy. In the two cases of the present study, a low density focus was observed in the CT scans. As the disease progresses, spot-like or irregular calcification may be observed in the focus or at the edge. In a study of 19 cases of abdominal TB, 50% of CT scans identified renal calcification and ~75% of renal tuberculous involvement was unilateral (10). Expansion of the pelvis, calices and ureter may be observed in advanced renal TB (11). In the second case of the present study, the patient presented with gross hematuria. The expansion of the pelvis, calices and ureter was easy to misdiagnosis as kidney calculi. Characteristic lobar calcification is often observed in end-stage TB (tuberculous autonephrectomy) (11).

In conclusion, the clinical and CT manifestations of renal TB are varied and may be misdiagnosed. In cases where there is no response to the usual antibiotic treatment in patients with fever or gross hematuria, TB should be suspected.
